# Colour appearance and compensation in the near periphery

**DOI:** 10.1098/rspb.2009.1832

**Published:** 2010-02-10

**Authors:** Michael A. Webster, Kimberley Halen, Andrew J. Meyers, Patricia Winkler, John S. Werner

**Affiliations:** 1 Department of Psychology, University of Nevada, Reno, NV, USA; 2 Department of Ophthalmology and Vision Science, Physiology and Behavior, University of California, Davis, CA, USA; 3 Department of Neurobiology, Physiology and Behavior, University of California, Davis, CA, USA

**Keywords:** colour, periphery, constancy

## Abstract

The spectral sensitivity of the visual system varies markedly between the fovea and surrounding periphery owing in part to the rapid fall in macular pigment density with eccentricity. We examined how colour appearance changes between the fovea and near periphery (8°) by measuring achromatic loci and the loci of unique and binary hues. Chosen colours remained much more similar at the two locations than predicted by the change in spectral sensitivity. Compensation for white may reflect long-term gain changes within the cones that equate sensitivity for the local average stimulus in the fovea and periphery. However, adjusting only to the average stimulus cannot correct for all of the effects of a spectral sensitivity change, and predicts differences in colour percepts between the fovea and periphery that were not observed. The similarities in hue percepts at 0 and 8° thus suggest that additional processes help compensate colour appearance to maintain constancy in the near periphery. We model the results of previous studies to show that similar adjustments are implied by age-related changes in lens pigment, and to show that these adjustments are consistent with previous measurements of peripheral colour appearance based on hue cancellation.

## 1. Introduction

To provide stable percepts of the properties of lights and surfaces, colour appearance must be compensated for the context in which objects are seen. Most studies of colour constancy have focused on contextual changes in the stimulus, such as discounting variations in illumination (Smithson 2005). However, a further important contextual effect confronting the visual system is intrinsic variations in the observer. For example, ageing is accompanied by large neural and optical changes, including increasing brunescence of the crystalline lens (Werner *et al*. 1990). If colour appearance were not compensated for these losses, then the same stimuli would look very different to younger and older observers. Yet, many properties of colour appearance instead remain stable across the lifespan. For example, the stimuli that appear white or a unique hue (pure blue, green, yellow or red) show little variation with age, suggesting that visual coding is continuously recalibrated to discount changes in sensitivity over time (Schefrin & Werner 1990; Werner & Schefrin 1993; Hardy *et al*. 2005). Consistent with this recalibration, achromatic settings also exhibit gradual but nearly complete recovery after the lens is replaced in cataract surgery (Delahunt *et al*. 2004).

We explored the effects of sensitivity changes across space on colour appearance, which arise from spatial variations in the retina. These include changes in macular pigment (Snodderly *et al*. 1984), photoreceptor morphology and photopigment density (Pokorny & Smith 1976; Marcos *et al*. 1997), absolute and relative density of different photoreceptors (Curcio *et al*. 1990, 1991) and types and number of different classes of bipolar and ganglion cells (Curcio & Allen 1990; Martin *et al*. 2001; Diller *et al*. 2004; Solomon *et al*. 2005).

Many previous psychophysical studies have examined how colour vision changes in the periphery. Chromatic sensitivity declines markedly with eccentricity (Moreland 1972) and is more rapid for chromatic contrasts detected by the L and M cones (Mullen & Kingdom 2002; Mullen *et al*. 2005), which may account for suggestions that the retina is characterized by ‘zones’ of chromatic sensitivity, with the red–green dimension restricted to more central regions (Moreland 1972; Sakurai *et al*. 2003). Above threshold, stimuli in the periphery appear less saturated, and require increasingly larger sizes or ‘perceptive fields’ to reach asymptotic levels (Abramov *et al*. 1991). Moreover, as eccentricity increases, there are again selective losses in perceived reddish–greenish contrasts so that hues are shifted towards bluish–yellowish directions (Parry *et al*. 2006; McKeefry *et al*. 2007). Finally, peripheral colour vision also changes through the influence of rods on hue percepts (Stabell & Stabell 1976; Buck *et al*. 1998; Nerger *et al*. 1998). Collectively, these effects demonstrate that changes in many aspects of colour vision tend to track the sensitivity limits imposed by physiological variations with retinal location. Indeed, studies of colour vision in the periphery have often been aimed at testing predictions about sensitivity and perception from identified properties of retinal coding, or have tried to infer properties of the neural substrate from behavioural changes. However, it remains unclear to what extent appearance is compensated for these sensitivity variations in order to promote colour constancy across the visual field.

We examined how colour appearance differs between the fovea and a nearby peripheral eccentricity of 8°. Over this range, there are again large changes in receptor distributions and their properties. Moreover, spectral sensitivity at the two loci can vary markedly because of changes in macular pigment density, which is highest in the central fovea and declines precipitously with eccentricity, reaching an asymptotic density at approximately 5° (Snodderly *et al*. 1984; Werner *et al*. 1987; Wooten & Hammond 2005). The macular pigment peaks in absorbance near 460 nm and absorbs minimally beyond approximately 540 nm, and thus selectively screens shorter wavelengths (Bone & Sparrock 1971). Sensitivity to short wavelengths is thus substantially reduced in the fovea relative to surrounding areas.

How colour appearance is affected by macular pigment screening remains uncertain. That appearance might be largely compensated for the sensitivity variation was first noted by Maxwell (1860), who suggested that ‘the reason why white light does not appear yellow in consequence, is that this absorbing action is constant, and we reckon as white the *mean* of all the colours we are accustomed to see’. Many reports have documented that macular screening or ‘Maxwell's spot’ is normally visible as an entoptic image only under uncharacteristic stimulation (e.g. flicker or narrowband mixtures; e.g. Nussbaum *et al*. 1981). A uniform appearance despite the macular variation could in principle occur from filling in across space (Magnussen *et al*. 2004; Beer *et al*. 2005; Balas & Sinha 2007). However, recent studies have found that even for isolated patches the stimulus that appears white does not significantly change between the fovea and surrounding regions (Beer *et al*. 2005; Webster & Leonard 2008). This suggests that the cones or their pathways are each adjusted for the difference in spectral sensitivity at the two loci so that the response to the same stimulus appears achromatic. These adjustments could reflect long-term gain changes in the cones that calibrate colour appearance for the chromatic properties of the environment (Pokorny & Smith 1977; Mollon 1982; Werner & Schefrin 1993), similar to the short-term adjustments, known as von Kries adaptation, which rescale sensitivity to discount average changes in the illuminant. If this renormalization takes place at the level of the photoreceptors, then all post-receptoral channels should be affected. Yet, studies using the method of hue cancellation—in which the hue of test lights is nulled by adding lights with opposing hues—have instead reported compensation for macular pigment only within the blue–yellow dimension of colour appearance (Hibino 1992; Stringham *et al*. 2006; Stringham & Hammond 2007). That is, blue–yellow nulls correspond to the same external stimulus and thus different cone catches in the fovea and periphery, while red–green nulls are instead predicted by the same quantal absorptions and thus require different stimuli.

We examined changes in colour appearance by measuring both achromatic loci and perceived hues for a wide range of stimuli. Confirming previous results, we find nearly complete compensation for white. This suggests that all three cone types are recalibrated for the change in spectral sensitivity, and we reanalyse the results of hue cancellation experiments to show that they are also consistent with gain changes in all cone types. We further show that the perceived hue of both broad and narrowband spectra remains similar at the 0 and 8° loci. This similarity cannot be accounted for by gain changes alone—since these adjust only to the average difference in sensitivity and thus cannot undo all of the effects of macular screening—and therefore implies additional compensatory calibrations of colour coding. We reanalyse previous measurements to show that adjustments beyond von Kries scaling are also necessary to explain the stability of unique hues despite changes in lens pigment density with ageing. Our results thus point to at least two distinct processes that might help promote colour constancy despite spatial variations in macular pigment or temporal variations in lens pigment within the observer.

## 2. Material and methods

### (a) Stimuli

Most stimuli were presented on a Sony 20SE CRT controlled by a Cambridge Research Systems VSG card and calibrated with a PhotoResearch PR650 spectroradiometer. Observers viewed the monitor from a distance of 114 cm through a black booth. Stimuli were uniform 2° squares shown at the centre of the monitor. Subjects viewed the fields directly for foveal settings, or while fixating a dim (5 cd m^−2^) grey cross at an eccentricity of 8°.

Narrowband stimuli were created with an Optronics Laboratories OL490 Agile Light Source, which filters light from a xenon source through a programmable DLP chip to create user-specified spectra. The spectra were displayed through the exit aperture of an integrating sphere as a 2° uniform disc on a black background. Stimuli were viewed directly from a distance of 143 cm or while fixating a dim white spot at an eccentricity of 8°.

### (b) Achromatic loci

Achromatic loci were measured with a method of adjustment. Observers dark adapted for 2 min and then viewed a repeating cycle of the test field flashed on for 0.5 s and off for 3 s. The field chromaticity was adjusted to appear achromatic by using buttons to vary colour along either axis of the1976 CIE u′v′uniform colour space. Stimuli had a constant photometric luminance of 25 cd m^−2^. Individual results are based on the mean of 12 settings from two sessions. Ten colour-normal observers were tested, including three authors and seven undergraduates. Protocols were approved by the university's IRB.

### (c) Hue angles

Hue angles were measured for stimuli presented in the same field by varying chromaticity around a circle of a fixed radius relative to the chromaticity of equal-energy white. The circle was defined within a version of the MacLeod–Boynton chromaticity diagram (MacLeod & Boynton 1979) centred on equal-energy white and scaled to roughly equate the perceived contrast along the *r* and *b* axes:
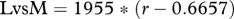

and
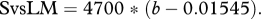



Scaled stimuli had a constant contrast of 100 and a constant luminance of 25 cd m^−2^. The field was again flashed on for 0.5 s, while the hue angle was varied in a staircase to estimate the angle corresponding to one of the four unique hues (blue, green, yellow or red), or one of the four binary hues defined by equal perceived mixture of adjacent unique hues. On each presentation, subjects made a 2-alternative forced choice (2AFC) response to indicate the field's colour (e.g. responding ‘too reddish’ or ‘too greenish’ for unique yellow), with the mean angle estimated from the final 10 staircase reversals (Webster *et al*. 2000). Observers made two settings for each hue at the foveal or 8° locations. For a small fraction of repetitions (3%), the angles differed by more than 30° and were excluded as unreliable estimates. Settings were made under adaptation to the dark background or to an isoluminant grey background (chromaticity of illuminant E).

### (d) Spectral hues

Spectral hues were measured for narrowband stimuli that had a Gaussian profile with a bandwidth (full width at half height) of 25 nm. A 2AFC staircase varied the chromaticity to determine the peak wavelength for the same target hues with the exception of unique red, which is extraspectral. Two repeated settings were measured for each hue and eccentricity.

### (e) Spectral sensitivity

Spectral sensitivity differences at the two loci were estimated for seven observers from minimum motion nulls (Beer *et al*. 2005; West & Mellerio 2005). The field was a 2° square displaying a 1 cycle per degree squarewave drifted at 2 Hz. The chromatic grating varied along the SvsLM axis over ±80 units. Differences in the motion nulls were fit with a template for the macular pigment to determine the relative density difference. Estimated densities ranged from 0 to 0.75 with a mean of 0.37. This is smaller than the differences typically reported between the fovea and periphery and may be due to the larger field size in the present measurements, since there is substantial fall-off in pigment density even by 1° of visual angle (Werner *et al*. 1987; Wooten & Hammond 2005).

## 3. Results

### (a) Achromatic settings

**Figure 1.**
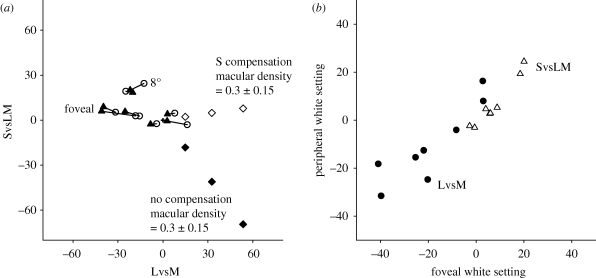
(*a*) Achromatic settings for eight observers at the fovea (open circles) or 8° (filled triangles). Lines connect settings for each observer. Filled diamonds: predicted change in periphery assuming no compensation for macular screening and a pigment density difference of 0.15, 0.3 or 0.45. Open diamonds: predictions if only the S cones are compensated. (*b*) LvsM (filled circles) or SvsLM (open triangles) coordinates in the fovea or periphery.

The first measurements replicated studies of white settings in the fovea and periphery (Beer *et al*. 2005; Webster & Leonard 2008). Figure 1 compares these settings to the differences predicted if there were no compensation for macular pigment or compensation only within S cones, based on adjusting the cone sensitivities (Smith & Pokorny 1975) by the macular pigment transmittance function (Bone & Sparrock 1971) scaled for a peak density of 0.3 ± 0.15 (consistent with the average difference for our observers). The foveal white point was assumed to correspond to illuminant E, and the monitor chromaticity required to generate the same cone ratios in the periphery was then calculated either without renormalizing the cones or renormalizing the S cones so that its mean response to an equal-energy spectrum equaled the foveal response.

A density difference of 0.3 predicts shifts of approximately 30 units between the foveal and peripheral settings along the LvsM axis (if only S cones are compensated) or both axes if there is no compensation. This is substantially larger than the observed differences. For the LvsM axis, the shift averaged 8 units, which was however significant (*t*(7) = 3.06, *p* = 0.018), while along the S axis the mean difference was less than 1 (*t*(7) = 0.76, *p* = 0.47). This suggests that there is substantial compensation for macular screening within all three cone classes, consistent with previous findings (Beer *et al*. 2005; Webster & Leonard 2008).

The stimulus perceived as white varied considerably across observers, yet comparatively little within each observer, resulting in high correlations between the foveal and peripheral settings for both the LvsM (*r* = 0.89) and SvsLM (*r* = 0.97) axes (figure 1*b*). Similarly, the variance within observers, based on the differences at 0 and 8°, was substantially lower than the differences between observers, based on the average of each observers' settings at 0 and 8°, for both LvsM (σ^2^_within_ = 7.8 versus σ^2^_between_ = 16.1; *F*(7) = 0.23, *p* = 0.037) and SvsLM (σ^2^_within_ = 2.8 versus σ^2^_between_ = 9.0; *F*(7) = 0.095, *p* = 0.003). Interobserver differences could potentially arise if individuals adopt different criteria for white or if they are exposed and thus adapted to environments with different average spectra (Webster & Leonard 2008). But whatever is defining the achromatic point, it leads to very similar settings at the two retinal locations and thus to nearly complete compensation for the sensitivity variations within the observer.

### (b) Unique and binary hues

#### (i) Hue angles

We next examined how hue percepts differed between the fovea and 8°.

**Figure 2.**
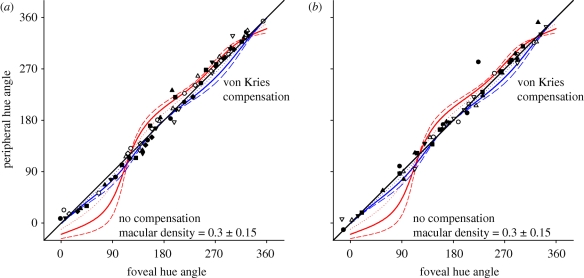
(*a*) Hue angles chosen by nine observers (indicated by symbols) for unique and binary hues in the fovea or periphery (hue settings on black background). Contours show settings predicted if there is no compensation for macular pigment (red), or if compensation adjusts only to the average spectral difference (blue), assuming macular pigment densities of 0.15 (dotted), 0.3 (solid) or 0.45 (dashed). (*b*) Settings for seven observers repeated on a grey background.

Figure 2 compares the stimulus angles chosen as unique or binary hues at the two loci. Note that these angles fill much of the entire circle of hues, indicating that like the white settings there were substantial individual differences in the hue loci (Webster *et al*. 2000). However, the hue settings at the fovea and 8° again remained similar, suggesting that there was little difference in appearance across locations despite the sensitivity differences. Mean settings did not significantly differ from perfect constancy (*t*(65) = 1.34, n.s.). (By ‘perfect constancy’ we mean identical hue settings at the two loci. More generally, constancy must fail because the loci will differ in metamerism.) In contrast, differences between the foveal and peripheral hue angles were significantly smaller than predicted by a conservative estimate of an average pigment density difference of 0.3 (*t*(65) = 7.19, *p* < 0.001). Moreover, fits to the observed hues by varying the macular pigment density were best for a density of 0.04, again close to the zero density differences predicted by perfect constancy.

Notably, the hue percepts also showed less difference than would be predicted if compensation only involved renormalizing the cones for the average spectral differences in the fovea and periphery. Rescaling the cones for the average spectrum completely corrects the white point, yet this correction in turn distorts the cone ratios for other spectra (in the same way that von Kries adaptation can correct for average changes in illumination, but not for all effects of an illuminant on colour appearance; Worthey & Brill 1986). These scaling effects are shown by the curves labelled von Kries compensation. The observed hue angles again significantly differ from the predictions of von Kries adaptation assuming a macular density of 0.3 (*t*(65) = 5.86, *p* < 0.001), and in this case, the best-fitting density was instead slightly negative (−0.06), and thus much closer to the perfect constancy (zero density) prediction. This suggests that the degree of constancy found for the peripheral settings cannot be explained by gain changes in the cones alone, and instead implicates an additional adjustment.

The settings on dark backgrounds required judging the hue of stimuli with a strong luminance increment (Abramov *et al*. 1992). The notion of a hue ‘angle’ in this case is vague because this angle is presumably relative to each observer's intrinsic white point. During testing, we also observed that under these conditions bluish hue angles were strongly and selectively reduced in apparent saturation, and thus became difficult to judge.

To better control for the mean adaptation level in the fovea and periphery and thus better isolate other potential factors in the hue settings, we repeated settings for the same test stimuli presented on the neutral grey background (figure 2*b*). The test chromaticity was thus an isoluminant exchange from the adapting field and was delimited by narrow black borders. Hue judgments remained similar to the settings on the black background (and bluish tests now appeared similar in saturation to other hue angles). Thus perceived hues again showed strong constancy between the fovea and periphery. Average settings did not differ from perfect constancy (*t*(52) = 1.45, n.s.), but were significantly different from the change predicted by the 0.3 macular pigment density (*t*(52) = 7.17, *p* < 0.001) and were best fit by a predicted density difference of 0.02. Note that, in this condition, white settings were biased to be similar in the fovea and periphery—by adapting observers to the same background at both locations—and thus gain changes in the cones were very likely to be actually adjusted for the sensitivity difference (as opposed to black backgrounds where this adjustment was instead inferred from the white settings). Yet, the hue percepts again significantly deviated from the settings predicted by these gain changes (*t*(52) = 5.75, *p* < 0.001). These results thus further implicate a form of compensation beyond spatially local gain changes that helps maintain colour appearance at different points across the central retina.

The common grey background presumably reduced differences in the adaptation states between observers and thus should have reduced the interobserver variance in achromatic settings (Webster & Leonard 2008). However, there was little evidence for systematic differences in the hue percepts under either adapting condition, again suggesting that the two retinal loci were already pre-adjusted to a similar state. The grey background did reduce *within*-observer variance in hue settings between the fovea and periphery (σ^2^ = 133 on black background versus 72 on grey, *F* = 0.54, *p* = 0.016). Yet, normalizing observers to a common white did not significantly reduce the *between*-observer variability in unique or binary hues at either location. Thus, individual differences in hue settings appear to depend little on differences in observers' intrinsic white points.

#### (ii) Narrowband stimuli

**Figure 3.**
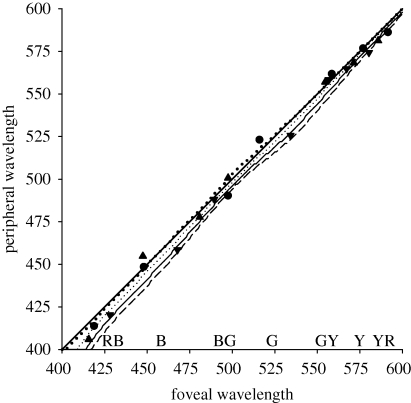
Wavelengths chosen as unique or binary hues at the fovea or 8° for three observers (symbols). Lines show shifts predicted if there is no compensation for macular pigment (bold dotted line), or compensation only for the average spectral difference for pigment densities of 0.3 ± 0.15 (dotted, solid or dashed lines).

In further measurements, we compared hues for narrowband stimuli instead of the more broadband monitor spectra. In this case, a change in macular pigment should have little effect on the settings, since—to the extent that the spectra approximate single wavelengths—this filtering changes only the intensity and not the shape of the spectra. However, differences are again predicted between the fovea and periphery if the cones at both locations are adjusted for the average difference in macular screening, because this requires different gain changes in the L, M and S cones and thus alters the cone ratios for narrowband stimuli. However, the results for these conditions did not clearly distinguish between the different predictions (figure 3).

### (c) Compensation for lens pigment

In further analyses, we turned to modelling results of previous studies to examine whether they were consistent with the patterns of compensation we observed for macular pigment screening. To further test for compensation beyond adjusting to the average colour, we first analysed previous results of the effects of lens pigment on colour appearance. The density of the lens pigment steadily increases with age and like macular pigment selectively screens shorter wavelengths (Werner *et al*. 1990). Despite these sensitivity changes, both achromatic settings and unique hues remain largely invariant with age (Schefrin & Werner 1990; Werner & Schefrin 1993). White settings could again be compensated for increasing lens pigment by renormalizing the cone sensitivities for the average colour signal, yet, as with macular pigment, these adjustments will not equate the ratio of cone responses to narrowband stimuli.

To assess these effects, we used estimates of age variations in lens transmittance (Pokorny *et al*. 1987) and then calculated the wavelengths that model observers at different ages would select to produce the same cone ratios relative to the age of 12 years. For each age, the cones were normalized so that responses were the same for a flat spectrum, predicting the observed constancy of white settings across age (Werner & Schefrin 1993). Stimuli were again modelled as Gaussian spectra with a bandwidth of 25 nm, so that predictions were equivalent to those illustrated in figure 3 except that lens pigment varied instead of macular pigment.

**Figure 4.**
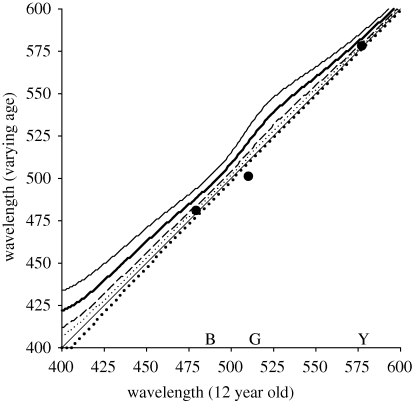
Predicted settings for narrowband hues in observers at different ages who differ only in lens pigment density. Lines: settings for observers age 12 (diagonal), 32 (dotted lines), 52 (dashed lines), 72 (bold dotted line) or 92 (solid lines). Circles: average projected differences between ages 12 and 72 in unique blue, green and yellow (Schefrin & Werner 1990).

Figure 4 plots predicted matching wavelengths between young and old observers. After normalizing the cones to equate white settings, older observers (with more lens pigment) should select longer wavelengths to preserve the cone ratios. Yet, these shifts are not observed in actual measurements (Schefrin & Werner 1990). Symbols plot the average estimated differences in unique blue, green or yellow for observers aged 12 or 72, corresponding to modelled lens densities at 400 nm of 1.36 or 2.41. Measured unique hues were invariant for blue and yellow, while shifting to shorter wavelengths for unique green by approximately 9 nm. Gain changes in the cones predict shifts of similar magnitude but in the opposite direction for blue and green, and a shift of approximately 5 nm for yellow. Thus, like the present results for macular pigment, the observed effects of lens pigment on colour appearance imply that additional processes beyond gain changes in the cones play a role in compensating colour appearance for physiological variations in observers.

### (d) Hue cancellation

In the final analysis, we re-examined why studies based on hue cancellation reached different conclusions about the influence of macular pigment on colour appearance. As noted, these studies reported complete compensation for macular pigment for the blue–yellow opponent dimension whereas little compensation for the red–green dimension, and attributed this to compensation only within S-cones or the blue–yellow pathway (Hibino 1992; Stringham *et al*. 2006; Stringham & Hammond 2007). The hue cancellation method involves nulling the hue in a test light by adding a primary light with the opposing hue, and thus determines the mixtures that appear ‘achromatic’ within each opponent axis. How can these findings be reconciled with the compensation we observed for both achromatic and hue settings?

To explore this, we asked how ‘blue–yellow’ and ‘red–green’ responses would be affected by gain changes in the cones, by using a simple linear model of colour opponency to define the opponent axes (Jameson & Hurvich 1968; Boynton 1979). Note that these perceptual axes differ from the LvsM and SvsLM cardinal axes defining the hue angles in figure 2 (Webster *et al*. 2000). The blue–yellow channel was formed by the difference in spectral sensitivity (Smith & Pokorny 1975) between S and the sum of L and M, weighted so that the difference was zero at a unique green of 512 nm. (Values for the unique hues vary widely, and we used values based on estimates from several studies; Kuehni 2004). The red–green channel was formed by the difference between L and M scaled to produce a unique yellow at 576 nm, and then adding S scaled to give a unique blue at 476 nm. Hue cancellation functions were then determined for the blue–yellow response by finding for each test wavelength the intensity of a ‘yellow’ (576 nm) or ‘blue’ (440 nm) primary needed to null the response. For red–green, the primaries were instead ‘green’ (500 nm) or ‘red’ (600 nm), consistent with previous studies (Stringham & Hammond 2007). Peripheral cones were adjusted assuming a macular pigment density difference of 0.3, and nulls were then determined for these new sensitivities or after renormalizing sensitivity so that the response to a flat spectrum was the same at both retinal locations.

**Figure 5.**
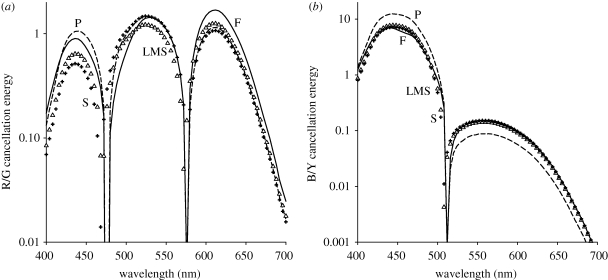
Predicted hue cancellation functions for (*a*) red–green or (*b*) blue–yellow. Absolute cancellation energy is shown for the fovea (solid curves) or periphery assuming no compensation (dashed curves), gain changes in all cones (triangles) or only in S (crosses), assuming a density difference of 0.3.

This simple model (Boynton 1979) does not correctly characterize many aspects of the perceptual opponent axes (which, for example, are nonlinear and require different cone combinations for different observers or for red versus green), but rather was used only to assess the specific question of how previous measures of hue cancellation functions might vary with the state of receptor adaptation. For red–green, gain changes in all cones or in S cones alone fail to correct for differences introduced by macular pigment (figure 5*a*). Thus, while the red–green response itself does not show compensation, this does not imply that cone inputs to this response are not adjusted for the difference in macular pigment. Rather, the red–green cancellation function is not a sensitive measure of these adjustments, because it does not strongly vary with them.

A different pattern is found for the blue–yellow function, where there is instead almost complete compensation for macular pigment (figure 5*b*). Moreover, similar compensation is predicted whether the renormalization is in all cones or restricted to S cones. This occurs because the peripheral change in the blue–yellow function primarily reflects the S response, so that renormalizing that response alone is sufficient to compensate for the change, whereas changes in L and M again have little influence.

The specific predictions depend on the choice of cone and opponent sensitivities and primaries, but the general pattern was robust over a range of assumed sensitivities and consistent with the major differences observed between the red–green and blue–yellow responses in the fovea and periphery (Hibino 1992; Stringham & Hammond 2007). This suggests that the results from hue cancellation and achromatic settings are not incompatible; instead, the gain changes in the cones implied by compensated white settings also account for much of the foveal versus near-peripheral differences in hue cancellation. Both techniques are consistent with an early visual adjustment for the average spectral shift introduced by differences in macular pigment.

## 4. Discussion

Similar to the stable colour appearance despite sensitivity changes in ageing, our results suggest that colour constancy is also maintained despite sensitivity changes across retinal location, at least within the central retina. Moreover, in both cases, at least two distinct adjustments may contribute to this constancy. These include gain changes in cone-specific pathways that scale sensitivity for the average stimulation, and a higher order adjustment that corrects perceived hue for the spectral filtering effects of the eye.

The notion that calibrating the white point might reflect an early and distinct adjustment is suggested by the finding that the stimulus that appears white in both the fovea and periphery is also the neutral stimulus for chromatic adaptation (Webster & Leonard 2008). That is, at either location, the stimulus that *looks* white does not induce a colour after-effect. This implies that the normalization occurs at or before the site of chromatic adaptation, which is largely cone-specific (Chichilnisky & Wandell 1995; Stockman *et al*. 2006). If achromatic signals were instead equated after adaptation, then equivalent adaptation in the fovea and periphery would instead require the same quantal catches, which correspond to perceptually different stimuli.

Changes in colour appearance with steady chromatic adaptation are generally well described by von Kries adaptation (Brainard & Wandell 1992; Webster & Mollon 1995; Wuerger 1996). Compensation for white could similarly occur if cones are adapted to the average stimulation they are exposed to. This adjustment must be spatially localized, and is consistent with the local adaptation pools found for photopic light adaptation (MacLeod *et al*. 1992). However, unlike von Kries adaptation, which is a rapid adjustment, equivalent foveal and peripheral white settings under dark adaptation imply a long-term calibration. Gradual adjustments of this kind are suggested by studies of achromatic loci in cataract patients, which take several months to return to near pre-surgery levels (Delahunt *et al*. 2004).

Gain adjustments will equate the perception of white only if the environment itself remains constant. This may be a reasonable approximation for the central visual angles we examined, but is less likely for larger eccentricities. Natural scenes spatially vary in their average spectra, for example, because of the sky and Earth (Webster *et al*. 2007). This raises the possibility that different retinal regions might be adapted in different ways leading to variations in achromatic loci across the visual field (Juricevic & Webster 2009).

It is well known that von Kries adaptation cannot undo all of the effects of an illuminant change, and thus could not provide complete colour constancy under different illuminants (Werner & Walraven 1982; D'Zmura & Lennie 1986; Worthey & Brill 1986; Webster & Mollon 1995). This limitation imposed by the spectral bandwidth of the receptors has also been noted in the colour vision of other species (Vorobyev *et al*. 1998; Dyer 1999). Similarly, photoreceptor gain changes alone cannot correct for all of the effects of screening filters like macular or lens pigment, and instead predict changes in the appearance of most spectra. Yet, observers' settings did not show these changes, suggesting that a second distinct adjustment helps to equate the perceived colour. The process underlying this calibration is uncertain, but requires mapping different cone ratios in the fovea and periphery onto the same hue sensations, and thus requires a post-receptoral adjustment that corrects for the spectral filtering of the eye. A functionally similar adjustment has been suggested by the finding that the hue of spectra tend to remain constant despite changes in spectral bandwidth, even though filtering by the eye's spectral sensitivity alters cone ratios for different bandwidths (Mizokami *et al*. 2006). It remains unclear whether the correction for spectral sensitivity limits reflects a form of adaptation versus learning to associate constant hues with the same surfaces viewed in different contexts (e.g. at different retinal locations).

The foveal and 8° retinal locations we tested differ on many dimensions, including inert screening pigments, cone and rod distributions and post-receptoral sampling. To the extent that colour vision can be compensated for these differences, caution should be exercised in relating measures of colour appearance to specific changes with eccentricity in retinal physiology or anatomy, for the characteristics of colour percepts have in any case been difficult to pin on a specific neural substrate (Brainard *et al*. 2000). On the other hand, as noted, there is overwhelming evidence that colour appearance does vary across the retina, and in particular, hue constancy clearly breaks down with increasing eccentricity (e.g. Parry *et al*. 2006; McKeefry *et al*. 2007). Thus, there are clear limits to this compensation. Further characterizing these peripheral appearance changes might provide insights into the factors that constrain the ability of the visual system to achieve perceptual constancy.

## References

[RSPB20091832C1] Abramov I. Gordon J. Chan H. 1991 Color appearance in the peripheral retina: effects of stimulus size. J. Opt. Soc. Am. A 8, 404–414 (doi:10.1364/JOSAA.8.000404) 2007915 10.1364/josaa.8.000404

[RSPB20091832C2] Abramov I. Gordon J. Chan H. 1992 Color appearance across the retina: effects of a white surround. J. Opt. Soc. Am. A 9, 195–202 (doi:10.1364/JOSAA.9.000195) 1542060 10.1364/josaa.9.000195

[RSPB20091832C3] Balas B. J. Sinha P. 2007 ‘Filling-in’ colour in natural scenes. Vis. Cogn. 15, 765–778 (doi:10.1080/13506280701295453)

[RSPB20091832C4] Beer D. Wortman J. Horwitz G. MacLeod D. 2005 Compensation of white for macular filtering [Abstract]. J. Vis. 5, 282a (doi:10.1167/5.8.282)

[RSPB20091832C5] Bone R. A. Sparrock J. M. 1971 Comparison of macular pigment densities in human eyes. Vis. Res. 11, 1057–1064 (doi:10.1016/0042-6989(71)90112-X) 5156776 10.1016/0042-6989(71)90112-x

[RSPB20091832C6] Boynton R. M. B. 1979 Human color vision New York, NY: Holt, Rinehart, and Winston

[RSPB20091832C7] Brainard D. H. Wandell B. A. 1992 Asymmetric color matching: how color appearance depends on the illuminant. J. Opt. Soc. Am. A 9, 1433–1448 (doi:10.1364/JOSAA.9.001433) 1527647 10.1364/josaa.9.001433

[RSPB20091832C8] Brainard D. H. Roorda A. Yamauchi Y. Calderone J. B. Metha A. Neitz M. Neitz J. Williams D. R. Jacobs G. H. 2000 Functional consequences of the relative numbers of L and M cones. J. Opt. Soc. Am. A Opt. Image. Sci. Vis. 17, 607–614 (doi:10.1364/JOSAA.17.000607) 10708042 10.1364/josaa.17.000607

[RSPB20091832C9] Buck S. L. Knight R. Fowler G. Hunt B. 1998 Rod influence on hue-scaling functions. Vis. Res. 38, 3259–3263 (doi:10.1016/S0042-6989(97)00436-7) 9893835 10.1016/s0042-6989(97)00436-7

[RSPB20091832C10] Chichilnisky E. J. Wandell B. A. 1995 Photoreceptor sensitivity changes explain color appearance shifts induced by large uniform backgrounds in dichoptic matching. Vis. Res. 35, 239–254 (doi:10.1016/0042-6989(94)00122-3) 7839619 10.1016/0042-6989(94)00122-3

[RSPB20091832C11] Curcio C. A. Allen K. A. 1990 Topography of ganglion cells in human retina. J. Comp. Neurol. 300, 5–25 (doi:10.1002/cne.903000103) 2229487 10.1002/cne.903000103

[RSPB20091832C12] Curcio C. A. Sloan K. R. Kalina R. E. Hendrickson A. E. 1990 Human photoreceptor topography. J. Comp. Neurol. 292, 497–523 (doi:10.1002/cne.902920402) 2324310 10.1002/cne.902920402

[RSPB20091832C13] Curcio C. A. Allen K. A. Sloan K. R. Lerea C. L. Hurley J. B. Klock I. B. Milam A. H. 1991 Distribution and morphology of human cone photoreceptors stained with anti-blue opsin. J. Comp. Neurol. 312, 610–624 (doi:10.1002/cne.903120411) 1722224 10.1002/cne.903120411

[RSPB20091832C14] Delahunt P. B. Webster M. A. Ma L. Werner J. S. 2004 Long-term renormalization of chromatic mechanisms following cataract surgery. Vis. Neurosci. 21, 301–307 (doi:10.1017/S0952523804213025) 15518204 10.1017/S0952523804213025 PMC2633455

[RSPB20091832C15] Diller L. Packer O. S. Verweij J. McMahon M. J. Williams D. R. Dacey D. M. 2004 L and M cone contributions to the midget and parasol ganglion cell receptive fields of macaque monkey retina. J. Neurosci. 24, 1079–1088 (doi:10.1523/JNEUROSCI.3828-03.2004) 14762126 10.1523/JNEUROSCI.3828-03.2004 PMC6793593

[RSPB20091832C16] Dyer A. G. 1999 Broad spectral sensitivities in the honeybee's photoreceptors limit colour constancy. J. Com. Physiol. A 185, 445–453 (doi:10.1007/s003590050405)

[RSPB20091832C17] D'Zmura M. Lennie P. 1986 Mechanisms of color constancy. J. Opt. Soc. Am. A 3, 1662–1672 3772628 10.1364/josaa.3.001662

[RSPB20091832C18] Hardy J. L. Frederick C. M. Kay P. Werner J. S. 2005 Color naming, lens aging, and grue: what the optics of the aging eye can teach us about color language. Psychol. Sci. 16, 321–327 (doi:10.1111/j.0956-7976.2005.01534.x) 15828980 10.1111/j.0956-7976.2005.01534.x PMC2586906

[RSPB20091832C19] Hibino H. 1992 Red–green and yellow–blue opponent-color responses as a function of retinal eccentricity. Vis. Res. 32, 1955–1964 (doi:10.1016/0042-6989(92)90055-N) 1287992 10.1016/0042-6989(92)90055-n

[RSPB20091832C20] Jameson D. Hurvich L. M. 1968 Opponent-response functions related to measured cone photopigments. J. Opt. Soc. Am. 58, 429–430

[RSPB20091832C21] Juricevic I. Webster M. A. 2009 Variations in normal color vision. V. Simulations of adaptation to natural color environments. Vis. Neurosci. 26, 133–145 (doi:10.1017/S0952523808080942) 19203426 10.1017/S0952523808080942 PMC2684467

[RSPB20091832C22] Kuehni R. G. 2004 Variability in unique hue selection: a surprising phenomenon. Color Res. Appl. 29, 158–162

[RSPB20091832C23] MacLeod D. I. Boynton R. M. 1979 Chromaticity diagram showing cone excitation by stimuli of equal luminance. J. Opt. Soc. Am. 69, 1183–1186 (doi:10.1364/JOSA.69.001183) 490231 10.1364/josa.69.001183

[RSPB20091832C24] MacLeod D. I. Williams D. R. Makous W. 1992 A visual nonlinearity fed by single cones. Vis. Res. 32, 347–363 (doi:10.1016/0042-6989(92)90144-8) 1574850 10.1016/0042-6989(92)90144-8

[RSPB20091832C25] Magnussen S. Spillmann L. Stürzel F. Werner J. S. 2004 Unveiling the foveal blue scotoma through an afterimage. Vis. Res. 44, 377–383 (doi:10.1016/j.visres.2003.09.023) 14659964 10.1016/j.visres.2003.09.023

[RSPB20091832C26] Marcos S. Tornow R. P. Elsner A. E. Navarro R. 1997 Foveal cone spacing and cone photopigment density difference: objective measurements in the same subjects. Vis. Res. 37, 1909–1915 (doi:10.1016/S0042-6989(96)00295-7) 9274776 10.1016/s0042-6989(96)00295-7

[RSPB20091832C27] Martin P. R. Lee B. B. White A. J. Solomon S. G. Ruttiger L. 2001 Chromatic sensitivity of ganglion cells in the peripheral primate retina. Nature 410, 933–936 (doi:10.1038/35073587) 11309618 10.1038/35073587

[RSPB20091832C28] Maxwell J. C. 1860 On the theory of compound colours, and the relations of the colours of the spectrum. Phil. Trans. R. Soc. 150, 57–84 (doi:10.1098/rstl.1860.0005)

[RSPB20091832C29] McKeefry D. J. Murray I. J. Parry N. R. 2007 Perceived shifts in saturation and hue of chromatic stimuli in the near peripheral retina. J. Opt. Soc. Am. A Opt. Image Sci. Vis. 24, 3168–3179 (doi:10.1364/JOSAA.24.003168) 17912307 10.1364/josaa.24.003168

[RSPB20091832C30] Mizokami Y. Werner J. S. Crognale M. A. Webster M. A. 2006 Nonlinearities in color coding: compensating color appearance for the eye's spectral sensitivity. J. Vis. 6, 996–1007 (doi:10.1167/6.9.12) 17083291 10.1167/6.9.12 PMC2583218

[RSPB20091832C31] Mollon J. D. 1982 Color vision. Annu. Rev. Psychol. 33, 41–85 (doi:10.1146/annurev.ps.33.020182.000353) 6977310 10.1146/annurev.ps.33.020182.000353

[RSPB20091832C32] Moreland J. D. 1972 Peripheral color vision. In Handbook of sensory physiology, vol. VII/4 (eds Jameson J. Hurvich L. M.), pp. 517–536 Berlin, Germany: Springer

[RSPB20091832C33] Mullen K. T. Kingdom F. A. 2002 Differential distributions of red–green and blue–yellow cone opponency across the visual field. Vis. Neurosci. 19, 109–118 12180855 10.1017/s0952523802191103

[RSPB20091832C34] Mullen K. T. Sakurai M. Chu W. 2005 Does L/M cone opponency disappear in human periphery? Perception 34, 951–959 (doi:10.1068/p5374) 16178149 10.1068/p5374

[RSPB20091832C35] Nerger J. L. Volbrecht V. J. Ayde C. J. Imhoff S. M. 1998 Effect of the S-cone mosaic and rods on red/green equilibria. J. Opt. Soc. Am. A Opt. Image Sci. Vis. 15, 2816–2826 (doi:10.1364/JOSAA.15.002816) 9803542 10.1364/josaa.15.002816

[RSPB20091832C36] Nussbaum J. J. Pruett R. C. Delori F. C. 1981 Macular yellow pigment: the first 200 years. Retina 1, 296–310 (doi:10.1097/00006982-198101040-00007) 6758089

[RSPB20091832C37] Parry N. R. McKeefry D. J. Murray I. J. 2006 Variant and invariant color perception in the near peripheral retina. J. Opt. Soc. Am. A Opt. Image Sci. Vis. 23, 1586–1597 (doi:10.1364/JOSAA.23.001586) 16783421 10.1364/josaa.23.001586

[RSPB20091832C38] Pokorny J. Smith V. C. 1976 Effect of field size on red–green color mixture equations. J. Opt. Soc. Am. 66, 705–708 (doi:10.1364/JOSA.66.000705) 978285 10.1364/josa.66.000705

[RSPB20091832C39] Pokorny J. Smith V. C. 1977 Evaluation of single-pigment shift model of anomalous trichromacy. J. Opt. Soc. Am. 67, 1196–1209 (doi:10.1364/JOSA.67.001196) 409816 10.1364/josa.67.001196

[RSPB20091832C40] Pokorny J. Smith V. C. Lutze M. 1987 Aging of the human lens. Appl. Opt. 26, 1437–1440 (doi:10.1364/AO.26.001437) 20454339 10.1364/AO.26.001437

[RSPB20091832C41] Sakurai M. Ayama M. Kumagai T. 2003 Color appearance in the entire visual field: color zone map based on the unique hue component. J. Opt. Soc. Am. A Opt. Image Sci. Vis. 20, 1997–2009 (doi:10.1364/JOSAA.20.001997) 14620327 10.1364/josaa.20.001997

[RSPB20091832C42] Schefrin B. E. Werner J. S. 1990 Loci of spectral unique hues throughout the life span. J. Opt. Soc. Am. A 7, 305–311 (doi:10.1364/JOSAA.7.000305) 2299452 10.1364/josaa.7.000305

[RSPB20091832C43] Smith V. C. Pokorny J. 1975 Spectral sensitivity of the foveal cone photopigments between 400 and 500 nm. Vis. Res. 15, 161–171 (doi:10.1016/0042-6989(75)90203-5) 1129973 10.1016/0042-6989(75)90203-5

[RSPB20091832C44] Smithson H. E. 2005 Sensory, computational and cognitive components of human colour constancy. Phil. Trans. R. Soc. B 360, 1329–1346 (doi:10.1098/rstb.2005.1633) 16147525 10.1098/rstb.2005.1633 PMC1609194

[RSPB20091832C45] Snodderly D. M. Auran J. D. Delori F. C. 1984 The macular pigment. II. Spatial distribution in primate retinas. Invest. Ophthalmol. Vis. Sci. 25, 674–685 6724837

[RSPB20091832C46] Solomon S. G. Lee B. B. White A. J. Ruttiger L. Martin P. R. 2005 Chromatic organization of ganglion cell receptive fields in the peripheral retina. J. Neurosci. 25, 4527–4539 (doi:10.1523/JNEUROSCI.3921-04.2005) 15872100 10.1523/JNEUROSCI.3921-04.2005 PMC6725021

[RSPB20091832C47] Stabell B. Stabell U. 1976 Rod and cone contribution to peripheral colour vision. Vis. Res. 16, 1099–1104 (doi:10.1016/0042-6989(76)90249-2) 969221 10.1016/0042-6989(76)90249-2

[RSPB20091832C48] Stockman A. Langendorfer M. Smithson H. E. Sharpe L. T. 2006 Human cone light adaptation: from behavioral measurements to molecular mechanisms. J. Vis. 6, 1194–1213 (doi:10.1167/6.11.5) 17209729 10.1167/6.11.5

[RSPB20091832C49] Stringham J. M. Hammond B. R. Jr 2007 Compensation for light loss due to filtering by macular pigment: relation to hue cancellation. Ophthal. Physiol. Opt. 27, 232–237 (doi:10.1111/j.1475-1313.2007.00462.x) 10.1111/j.1475-1313.2007.00462.x 17470235

[RSPB20091832C50] Stringham J. M. Hammond B. R. Wooten B. R. Snodderly D. M. 2006 Compensation for light loss resulting from filtering by macular pigment: relation to the S-cone pathway. Optom. Vis. Sci. 83, 887–894 (doi:10.1097/01.opx.0000249976.00534.2d) 17164681 10.1097/01.opx.0000249976.00534.2d

[RSPB20091832C51] Vorobyev M. Osorio D. Bennett A. T. D. Marshall N. J. Cuthill I. C. 1998 Tetrachromacy, oil droplets and bird plumage colours. J. Comp. Physiol. A 183, 621–633 (doi:10.1007/s003590050286) 9839454 10.1007/s003590050286

[RSPB20091832C52] Webster M. A. Leonard D. 2008 Adaptation and perceptual norms in color vision. J. Opt. Soc. Am. A Opt. Image Sci. Vis. 25, 2817–2825 (doi:10.1364/JOSAA.25.002817) 18978861 10.1364/josaa.25.002817 PMC2657039

[RSPB20091832C53] Webster M. A. Mollon J. D. 1995 Colour constancy influenced by contrast adaptation. Nature 373, 694–698 (doi:10.1038/373694a0) 7854451 10.1038/373694a0

[RSPB20091832C54] Webster M. A. Miyahara E. Malkoc G. Raker V. E. 2000 Variations in normal color vision. II. Unique hues. J. Opt. Soc. Am. A Opt. Image Sci. Vis. 17, 1545–1555 (doi:10.1364/JOSAA.17.001545) 10975364 10.1364/josaa.17.001545

[RSPB20091832C55] Webster M. A. Mizokami Y. Webster S. M. 2007 Seasonal variations in the color statistics of natural images. Network 18, 213–233 (doi:10.1080/09548980701654405) 17926193 10.1080/09548980701654405

[RSPB20091832C56] Werner J. S. Schefrin B. E. 1993 Loci of achromatic points throughout the life span. J. Opt. Soc. Am. A 10, 1509–1516 (doi:10.1364/JOSAA.10.001509) 8350147 10.1364/josaa.10.001509

[RSPB20091832C57] Werner J. S. Walraven J. 1982 Effect of chromatic adaptation on the achromatic locus: the role of contrast, luminance and background color. Vis. Res. 22, 929–943 (doi:10.1016/0042-6989(82)90029-3) 7135855 10.1016/0042-6989(82)90029-3

[RSPB20091832C58] Werner J. S. Donnelly S. K. Kliegl R. 1987 Aging and human macular pigment density. Appended with translations from the work of Max Schultze and Ewald Hering. Vis. Res. 27, 257–268 (doi:10.1016/0042-6989(87)90188-X) 3554735 10.1016/0042-6989(87)90188-x

[RSPB20091832C59] Werner J. S. Peterzell D. H. Scheetz A. J. 1990 Light, vision, and aging. Optom. Vis. Sci. 67, 214–229 (doi:10.1097/00006324-199003000-00013) 2181364 10.1097/00006324-199003000-00013

[RSPB20091832C60] West P. Mellerio J. 2005 An innovative instrument for the psychophysical measurement of macular pigment optical density using a CRT display. http://www.crsltd.com/research-topics/macular-pigment/index.html

[RSPB20091832C61] Wooten B. R. Hammond B. R. Jr 2005 Spectral absorbance and spatial distribution of macular pigment using heterochromatic flicker photometry. Optom. Vis. Sci. 82, 378–386 (doi:10.1097/01.OPX.0000162654.32112.A1) 15894914 10.1097/01.OPX.0000162654.32112.A1

[RSPB20091832C62] Worthey J. A. Brill M. H. 1986 Heuristic analysis of von Kries color constancy. J. Opt. Soc. Am. A 3, 1708–1712 (doi:10.1364/JOSAA.3.001708) 3772633 10.1364/josaa.3.001708

[RSPB20091832C63] Wuerger S. M. 1996 Color appearance changes resulting from iso-luminant chromatic adaptation. Vis. Res. 36, 3107–3118 (doi:10.1016/0042-6989(96)00057-0) 8917772 10.1016/0042-6989(96)00057-0

